# Trees in Winter

**Published:** 1874-03

**Authors:** 


### TREES IN WINTER.

A REMARKABLE paper has recently been contributed to a German magazine by Professor Mohr, showing not only that the sap does not freeze in trees and plants which live through hard winters, but also the reason why it does not freeze. He says that though water, as we generally see and understand it, freezes at thirty-two degrees, it does not do so when its particles are finely divided. Tropical plants have large cells, and these are the ones in which the sap freezes; but in plants with very small cells, in which the liquid particles are finely divided, there is no freezing of the liquids until after the structure has received injury of some sort. This is true, he says, of insects and insect pupæ. They never freeze; but cut one apart, soon after the humors solidify, and, on thawing, life dies.

